# Supplementary material for: Genome-Wide Analysis of the Amino Acid Permeases Gene Family in Wheat and TaAAP1 Enhanced Salt Tolerance by Accumulating Ethylene
Source: Int J Mol Sci. 2023 Sep 7;24(18):13800. doi: 10.3390/ijms241813800 (PMC10530925; doi:10.3390/ijms241813800)
Supplement: Supplementary file 1 [file ijms-24-13800-s001.zip › Figures S1-S3.pdf]

**Supplementary data**

**Table S1. General information of the 217 genes search by PF01490 in the WheatOmics database.**

**Table S2. The conserved domain of 70 putative amino acid permeases using Batch CD-search tools.**

**Table S3. Detailed information of the identified 51 *TaAAPs*.**

**Table S4. Primers used in this article.**

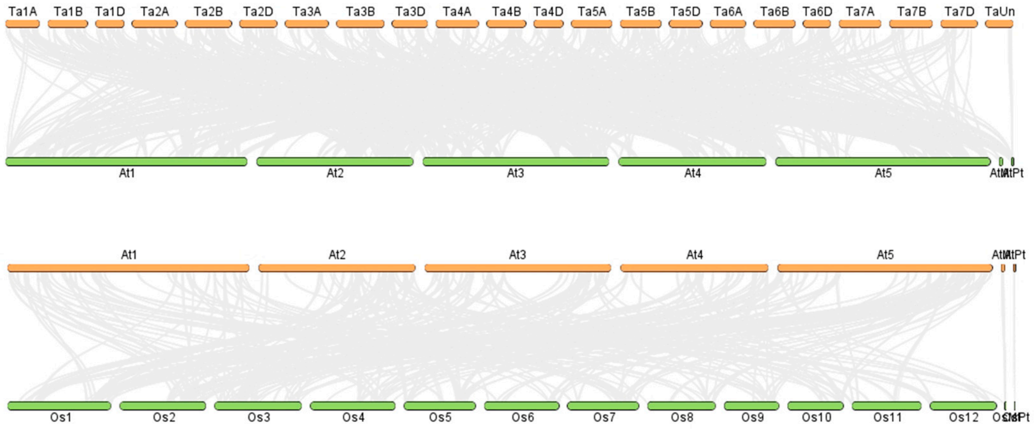


**Figure S1. Synteny analysis of the AAPs family in wheat and rice with *Arabidopsis*, respectively.**

The gray lines indicate collinear blocks in plant genomes.

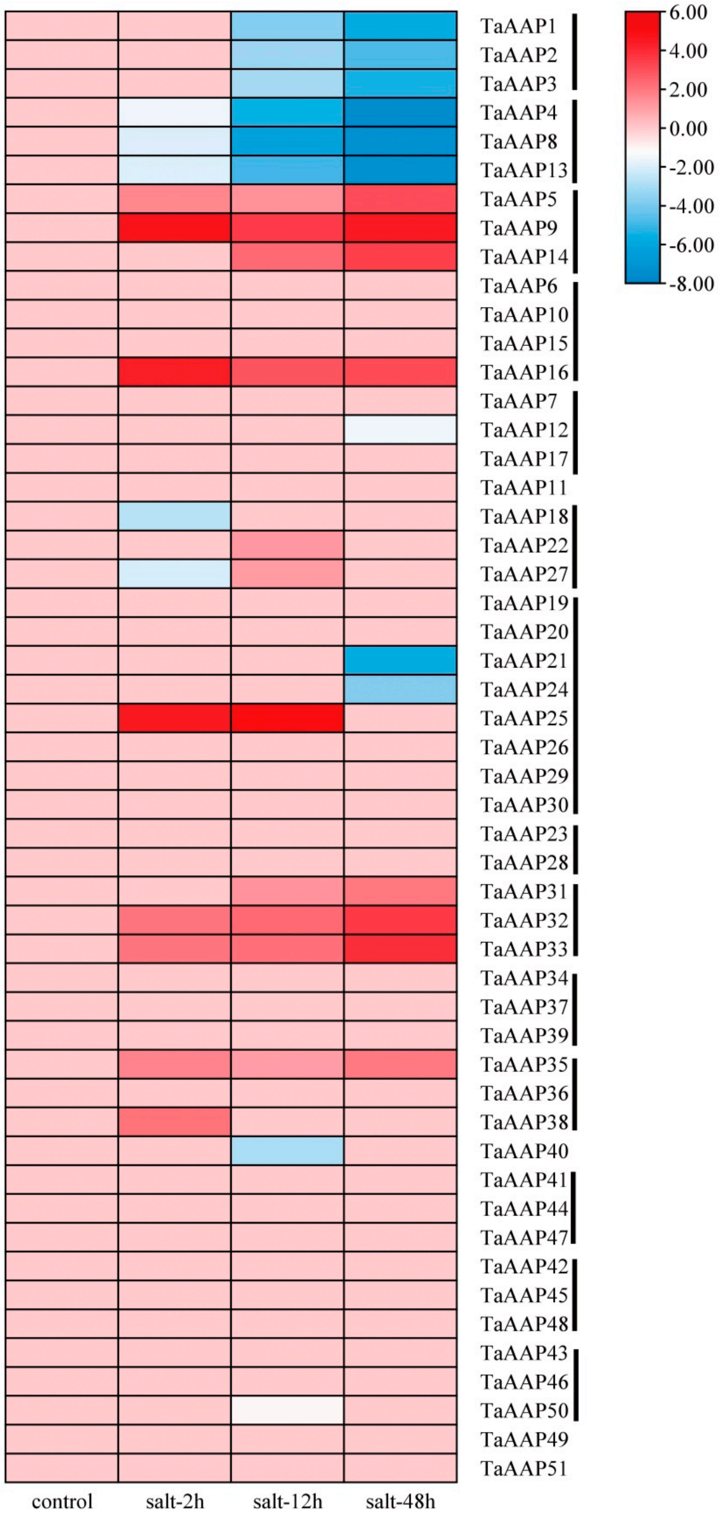


**Figure S2. Expression profiles of 51 *TaAAPs* genes in salt stress.**

Seven days old Jimai 22 seedling in 1/2 Hoagland solution with or without 200 mM NaCl for 0/2/12/48 hours, seedlings were harvested for RNA sequencing. Heat map is the expression level of *TaAAPs* genes under salinity stress.

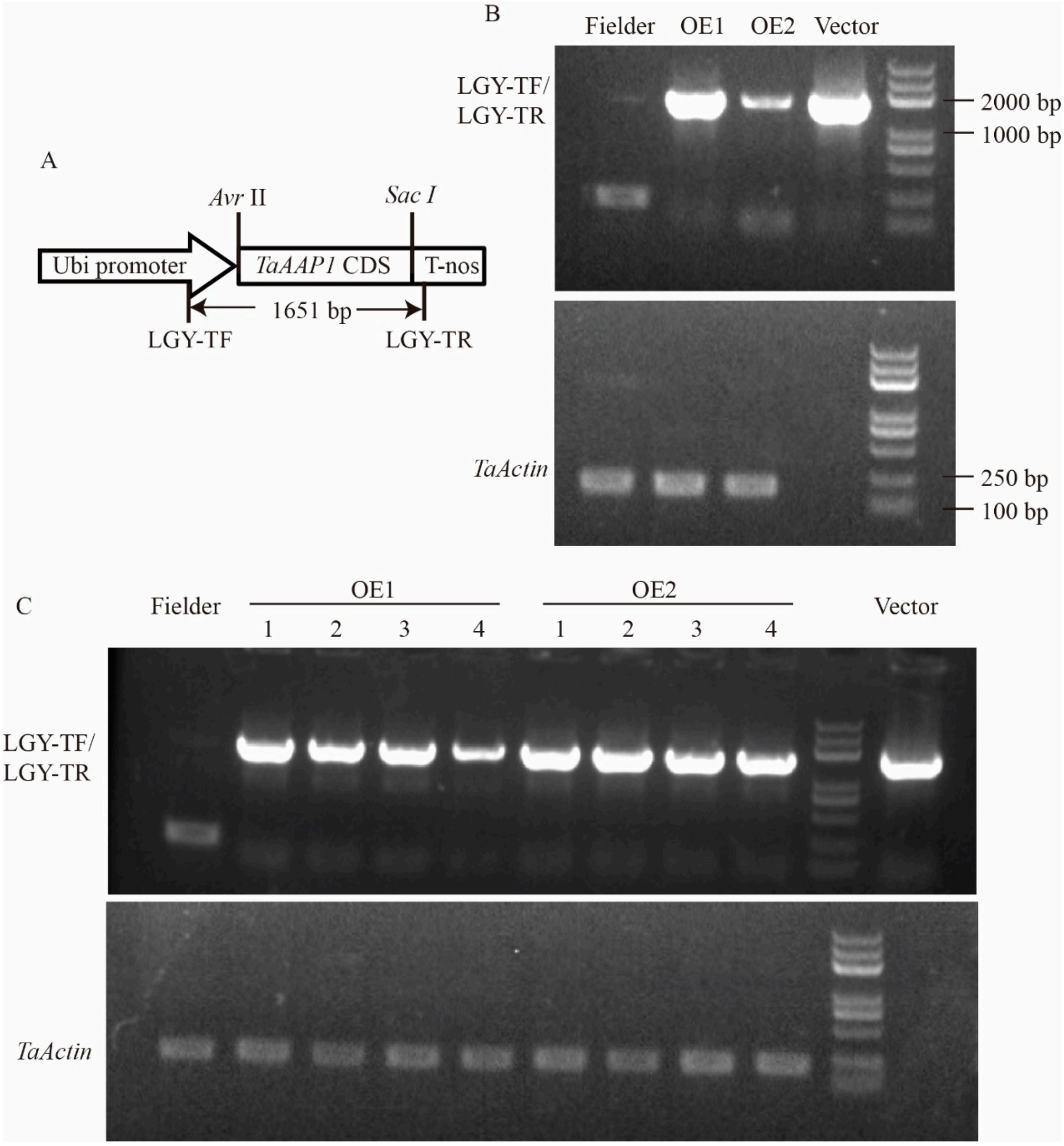


**Figure S3. Construction of pLGY-OE3-*TaAAP1* and molecular detection of *TaAAP1*-overexpressing transgenic wheat lines.**

(A) The CDS of *TaAAP1* was subcloned into pLGY-OE3 vector with *Avr* II and *Sac* I restriction enzyme sites. The transgenic plants were detected via PCR with primers LYG-TF and LGY-TR in T1 (B) and T2 (C). The PCR product was 1651 bp, *TaActin* was the reference gene, the vector as the positive control.
